# Inhibiting PHGDH with NCT-503 reroutes glucose-derived carbons into the TCA cycle, independently of its on-target effect

**DOI:** 10.1080/14756366.2021.1935917

**Published:** 2021-06-30

**Authors:** Birte Arlt, Guido Mastrobuoni, Jasmin Wuenschel, Kathy Astrahantseff, Angelika Eggert, Stefan Kempa, Hedwig E. Deubzer

**Affiliations:** a Department of Pediatric Hematology and Oncology, Charité – Universitätsmedizin Berlin, Berlin, Germany; b Neuroblastoma Research Group, Experimental and Clinical Research Center (ECRC) of the Charité and the Max-Delbrück-Center for Molecular Medicine (MDC) in the Helmholtz Association, Berlin, Germany; c Berlin Institute of Health (BIH), Berlin, Germany; d Integrative Proteomics and Metabolomics, Berlin Institute for Medical Systems Biology at the Max-Delbrück Center for Molecular Medicine in the Helmholtz Association, Berlin, Germany; e German Cancer Consortium (DKTK), Berlin, Germany; f German Cancer Research Center (DKFZ), Heidelberg, Germany

**Keywords:** Cancer cell metabolism, de novo serine synthesis pathway, citrate synthase, pulsed stable isotope-resolved metabolomics, thermal shift assay

## Abstract

The small-molecule inhibitor of phosphoglycerate dehydrogenase, NCT-503, reduces incorporation of glucose-derived carbons into serine *in vitro*. Here we describe an off-target effect of NCT-503 in neuroblastoma cell lines expressing divergent phosphoglycerate dehydrogenase (PHGDH) levels and single-cell clones with CRISPR-Cas9-directed PHGDH knockout or their respective wildtype controls. NCT-503 treatment strongly reduced synthesis of glucose-derived citrate in all cell models investigated compared to the inactive drug control and independent of PHGDH expression level. Incorporation of glucose-derived carbons entering the TCA cycle via pyruvate carboxylase was enhanced by NCT-503 treatment. The activity of citrate synthase was not altered by NCT-503 treatment. We also detected no change in the thermal stabilisation of citrate synthase in cellular thermal shift assays from NCT-503-treated cells. Thus, the direct cause of the observed off-target effect remains enigmatic. Our findings highlight off-target potential within a metabolic assessment of carbon usage in cells treated with the small-molecule inhibitor, NCT-503.

## Introduction

Neuroblastoma is the most common extracranial paediatric solid tumour that arises from precursor cells in the developing sympathetic nervous system. Amplification of the *MYCN* oncogene among other molecular features, increase neuroblastoma aggressiveness and risk for relapse^1^. Directly targeting MYCN remains a challenge due to its nuclear localisation, the lack of a ligand binding site and its diverse physiological functions in the maintenance of normal tissues^2^. Indirect approaches targeting metabolic alterations driven by high-level *MYCN* amplification recently identified an enhanced dependency of these cells on the serine-glycine one-carbon metabolic pathway^3^. This network generates one-carbon units that are utilised in various metabolic reactions, including nucleotide biosynthesis, methylation reactions and maintaining redox balance. Cells produce one-carbon units from serine, which can either be imported or synthesised *de novo* through the rate-limiting enzyme, phosphoglycerate dehydrogenase (PHGDH). It is well appreciated that the small-molecule PHGDH inhibitor, NCT-503, reduces production of glucose-derived serine and attenuates the growth of PHGDH-dependent cell lines and xenograft tumours^4^. In recent work, we have shown that NCT-503 reduces proliferation *in vitro* and initial tumour growth *in vivo* in *MYCN*-amplified neuroblastoma cells^5^. Here we assessed proliferation and applied pulsed stable isotope-resolved metabolomics utilising ^13^C-glucose labelling^6^ in cellular models for neuroblastoma expressing varying levels of the NCT-503 target, PHGDH, with the aim to assess the impact of NCT-503 treatment on central carbon metabolism.

## Materials and methods

### Cell culture

The BE(2)-C (RRID: CVCL_V006) cell line was obtained from ECACC (Salisbury, UK) and the Kelly (RRID: CVCL_2092) cell line from the DSMZ (Braunschweig, Germany). The SK-N-AS (RRID: CVCL_1700) cell line was kindly provided by J. Schulte (Charité, Berlin, Germany) and the SH-EP (RRID: CVCL_0524) cell line by L. Savelyeva (DKFZ, Heidelberg, Germany). CRISPR/Cas9-mediated *PHGDH* knockout-clones were generated as described^5^. In brief, the plasmids, px459_PH_ex4_80 (clone #11) or px459_PH_ex7_96 (clone #38), were transfected into BE(2)-C cells using the Effectene (Qiagen, Hilden, Germany) method according to the manufacturer’s directions. These plasmids encode for SpCas9 and a gRNA targeting *PHGDH* exon 4 (5′-TGCCGGAAGATCTTGCAAGA-3′) or *PHGDH* exon 7 (5′-CTTCATCGAAGCCGTCGCCT-3′). Control cells were transfected with the px459 vector, which expresses SpCas9 (pSpCas9(BB)-2A-Puro; #48139, Addgene, Watertown, MA, USA). After 24 h, 2 µg/ml puromycin (Thermo Fisher Scientific, Waltham, MA, USA) was added to the culture medium for 72 h to enrich for positively transfected cells. Thereafter, limited dilution assays were performed by seeding 0.5–1 cell per well into 96-well plates. All expandable single-cell clones were subjected to western blotting and pulsed stable isotope-resolved metabolomics coupled to GC-MS to identify PHGDH knockouts. Cell lines were maintained at 37 °C and 5% CO_2_ in DMEM (Thermo Fisher Scientific, Waltham, MA, USA) medium lacking glucose, glutamine, sodium pyruvate and phenol red and supplemented with 10% foetal calf serum (Merck, Darmstadt, Germany), 2.5 g/L glucose (Merck, Darmstadt, Germany) and 2 mM glutamine (Thermo Fisher Scientific, Waltham, MA, USA). Cell lines were maximally cultured up to passage 28. Cell lines were authenticated by high-throughput SNP-based assays^7^ and weekly monitored for mycoplasma infections using PlasmoTest™ (InvivoGen, San Diego, CA, USA) according to the manufacturer’s instructions.

### Drug source and preparation

*N*-(4,6-Dimethylpyridin-2-yl)-4-(4-(trifluoromethyl)benzyl)piperazine-1-carbothioamide, the NCT-503 small molecule inhibitor (SML1659, Sigma-Aldrich, St. Louis, MO, USA) and *N*-(4,6-dimethylpyridin-2-yl)-4-pyridin-4-ylpiperazine-1-carbothioamide, the inactive NCT-503 control (SML1671, Sigma-Aldrich, St. Louis, MO, USA) were first described by Pacold et al.^4^. The 4-trifluoromethyl substituent of NCT-503 was replaced with a 4-pyridinyl group resulting in the inactive NCT-503 control compound that did not inhibit PHGDH^4^, which was used as the inactive drug control in this study. NCT-503 and inactive NCT-503 control were dissolved in DMSO for 9.9 mM and 15.3 mM stock solutions, which were aliquoted and stored at 4 °C until use. Cells were treated with 10 µM of either compound diluted in culture medium.

### Western blotting

Cells were lysed for western blotting in buffer containing 20 mM Tris-HCl, 7 M urea, 0.01% Triton X-100, 100 mM DTT, 40 mM MgCl_2_ and Complete® protease inhibitor cocktail, and proteins separated by 12% SDS-PAGE then semi-dry blotted polyvinylidene difluoride membranes (Roche, Basel, Switzerland) probed with a mouse monoclonal antibody against GAPDH (MAB374, clone 6C5, Merck, Darmstadt, Germany, RRID:AB_2107445) and a rabbit polyclonal antibody against PHGDH (#13428, Cell Signalling, Danvers, MA, USA, RRID:AB_2750870). Band density was analysed using VisionCapt software, version 16.11a (Vilber Lourmat, Eberhardzell, Germany) on western blots, and results were normalised to the respective loading controls.

### Proliferation assay

Cell viability and number were measured with the VI-CELL-XR Cell Viability Analyser (Beckman Coulter, Brea, CA, USA) based on the Trypan blue exclusion method^8^.

### Pulsed stable isotope-resolved metabolomics and GC-MS analysis

Cells were seeded as indicated for labelling experiments, and medium change was performed 4 h before harvest^6^. A second medium change was performed 10 min before harvest to expose cells to media supplemented with either 2.5 g/L ^13^C-glucose or 2.5 g/L^12^C-glucose, the latter representing the natural mass isotopic distribution. Cells were washed with HEPES buffer (140 mM NaCl, 5 mM HEPES, pH 7.4) containing labelled or non-labelled glucose and quenched by adding 50% ice-cold methanol containing 2 µg/ml cinnamic acid (Merck, Darmstadt, Germany) as an internal control. Metabolite extraction was performed as described^9^. In brief, polar metabolites were extracted by methanol/chloroform/water extraction, centrifuged at 4000*g* for 10 min at 4 °C and dried in a vacuum concentrator overnight. For GC-MS measurement, extracts were dissolved in methoxyamine hydrochloride solution (40 mg/mL MeOX in pyrimidine) at 30 °C for 90 min with constant shaking followed by an incubation at 37 °C for 60 min with *N*-methyl-*N*-[trimethylsilyl] trifluoroacetamide containing an alkane mixture. Extracts were centrifuged at maximum speed for 10 min, and aliquots were transferred into glass vials for GC-MS measurement. Metabolite analysis was performed on a Q Exactive GC Orbitrap system (Thermo Fisher Scientific, Waltham, MA, USA). Samples were injected in 1:10 split mode with an injection volume of 1 µL in a temperature controlled injector (TriPlus RSH autosampler, Thermo Fisher Scientific, Waltham, MA, USA) with baffled glass liner (Thermo Fisher Scientific, Waltham, MA, USA). The injection temperature was kept at 80 °C for 15 s, then increased to 260 °C with a ramp of 7° C/s and kept at 260 °C for 3 min. Gas chromatographic separation was performed on a Thermo Trace1300 GC system (Thermo Fisher Scientific, Waltham, MA, USA), equipped with a TG-5SILMS column (30 m length, 0.25 mm inner diameter, 0.25 µm film thickness). Helium was used as carrier gas with a flow rate of 1.2 ml/min. Gas chromatography was performed with the following program: initial temperature of 67.5 °C for 2 min, 1st ramp to 120 °C with 7 °C/min, 2nd ramp to 200 °C with 9 °C/min, 3rd ramp to 330 °C with 12 °C/min and final hold at 350 °C for 6 min. Spectra were acquired in the range from 65 to 600 *m*/*z*, with a resolution of 30,000 (at *m*/*z* 200). GC-MS chromatograms were processed with Xcalibur Quan Browser 4.3 software, and the identity of the analysed metabolites was verified by standards as described^10^.

### Citrate synthase activity assay

Cells were treated for 24 h with 10 µM NCT-503 or inactive NCT-503 control, and then lysed in CelLytic M provided in the citrate synthase assay kit (CS0720, Sigma-Aldrich, St. Louis, MO, USA). Aliquots of whole-cell extract containing equal total protein amounts were used for the citrate synthase activity assay according to the manufacturer’s instructions. Total protein was assessed by colorimetric protein quantification based on the Bradford assay, ROTI®Quant (Carl Roth, Karlsruhe, Germany).

### Acetyl-coenzyme A assay

Cells were treated for 48 h with 10 µM NCT-503 or inactive NCT-503 control. Aliquots with equal numbers of cells were analysed by the acetyl-coenzyme A (acetyl-CoA) assay kit (MAK039, Sigma-Aldrich, St. Louis, MO, USA) according to the manufacturer’s instructions.

### Cellular thermal shift assay

Cells were treated for 48 h with 10 µM NCT-503 or inactive NCT-503 control, scraped from the culture plates using a policeman for the cellular thermal shift assay as previously described^11^ using a rabbit polyclonal antibody against PHGDH (#13428, Cell Signalling, Danvers, MA, USA, RRID:AB_2750870) and a rabbit monoclonal antibody against citrate synthase (#14309, Cell Signalling, Danvers, MA, USA, RRID:AB_2665545). Band density on western blots was analysed using VisionCapt software, version 16.11a (Vilber Lourmat, Eberhardzell, Germany), and data was normalised by setting the highest and lowest value in each dataset to 100% and 0%, respectively.

### Statistical analysis

Statistical analysis was performed using the GraphPad Prism (version 7, GraphPad Software, La Jolla, CA, USA) and R-studio (version 1.1.383) software packages with R version 3.4.0^12^. If not stated otherwise, a two-tailed Student’s *t*-test with Welch’s correction was applied to test significance of differences between testing groups.

## Results

### NCT-503 reduces viability of cells expressing low target enzyme levels

**Figure 1.**
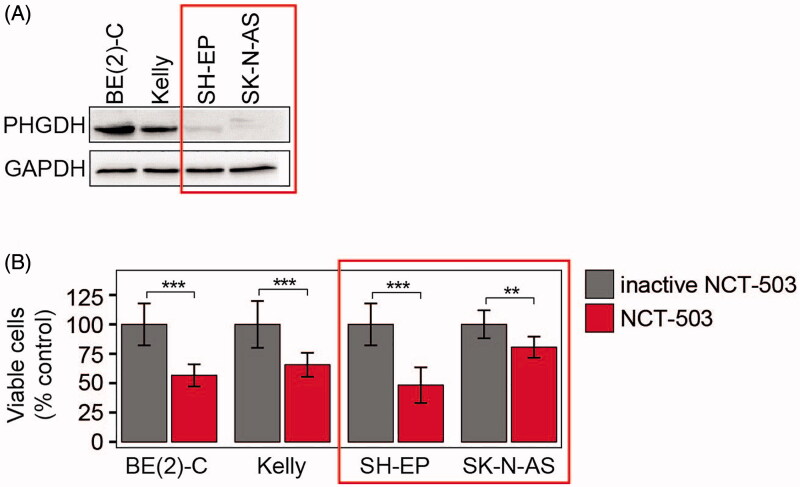
NCT-503 treatment inhibits proliferation of neuroblastoma cells with low target enzyme expression. (A) Western blot analysis of PHGDH expression in a panel of four neuroblastoma cell lines. GAPDH served as loading control. (B) Proliferation assay indicating the number of viable BE(2)-C, Kelly, SH-EP and SK-N-AS cells treated with 10 µM NCT-503 or inactive drug control (inactive NCT-503) for 96 h (mean ± SD, *n* = 3). ***p ≤ 0.001.

We have previously shown that NCT-503 treatment reduces proliferation of neuroblastoma cells expressing high PHGDH levels *in vitro* and initially reduces patient-derived neuroblastoma xenograft tumour volumes in mice^5^. By random observation, we noticed that NCT-503 treatment also attenuated proliferation of neuroblastoma cells expressing low PHGDH levels. We extended proliferation monitoring to a panel of two neuroblastoma cell lines each expressing either high-level (BE(2)-C and Kelly) or low-level (SH-EP and SK-N-AS) PHGDH (Figure 1(A)). NCT-503 treatment significantly decreased the number of viable cells in all four cell lines compared to cultures treated with the inactive drug control (Figure 1(B)). Viable BE(2)-C and SH-EP cells were reduced to 50–60% of controls and viable Kelly and SK-N-AS cells by 65–80% of controls, demonstrating no clear dependency on target expression level in the cell lines and a broad range of 20–50% reduction in viability in all cell lines tested. These data indicate that NCT-503 is effective in a PHGDH-independent manner in neuroblastoma cells, raising the possibility that some part of NCT-503 action may be caused by off-target effects.

### NCT-503 treatment reduces carbon flow into the TCA cycle

**Figure 2.**
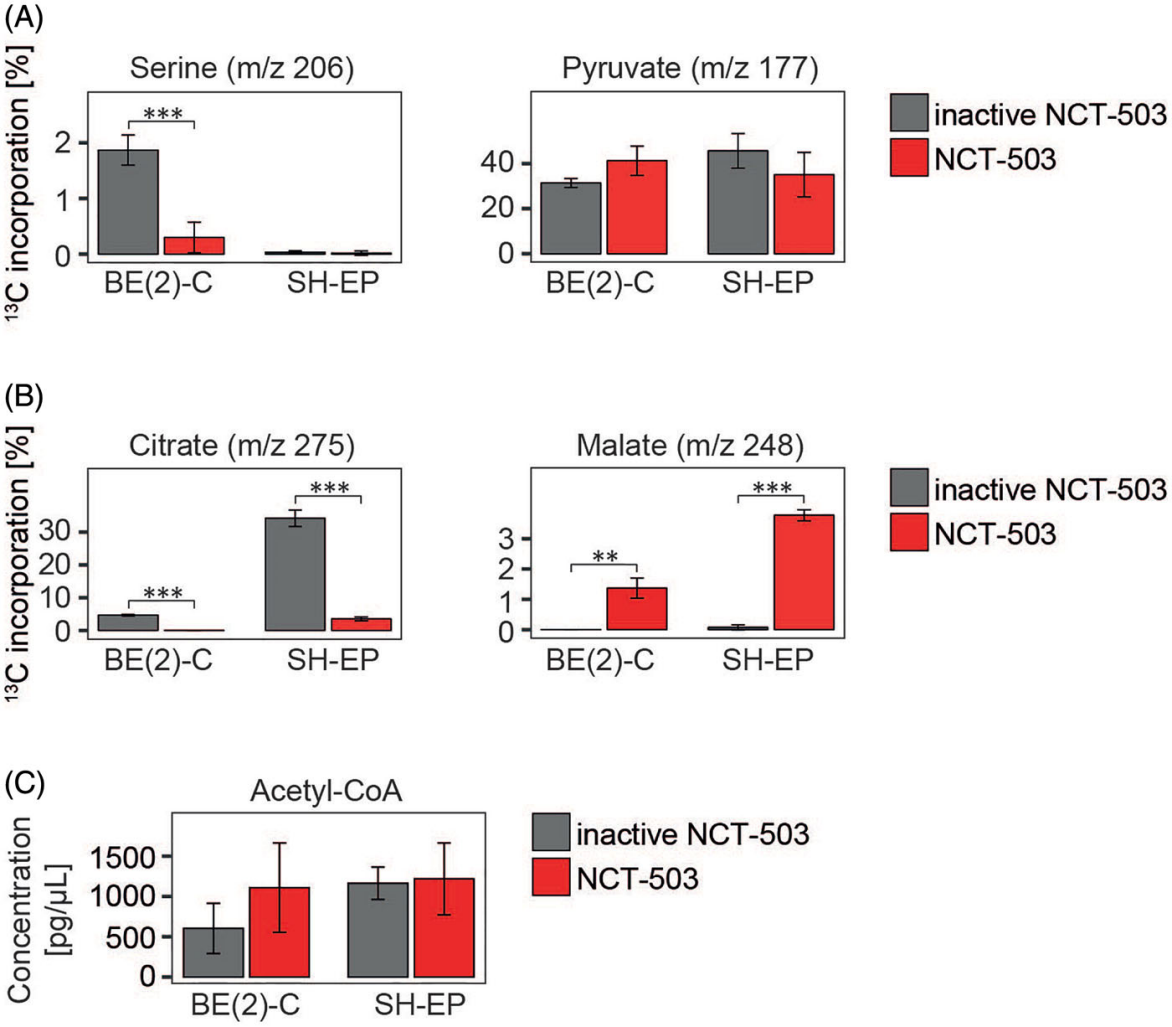
NCT-503 treatment reduces the carbon flow into citrate in the TCA cycle. BE(2)-C and SH-EP cells were seeded, cultured for 48 h with 10 µM NCT-503 or inactive drug control and maintained in medium containing fully labelled ^13^C-glucose (13 mM) for 10 min before harvest. Shown are the percentages of ^13^C-label incorporation into serine and pyruvate (A), citrate and malate (B) and the quantification of acetyl-CoA levels (C). **p ≤ 0.01; ***p ≤ 0.001.

Since PHGDH catalyses the rate-limiting step in serine synthesis, we performed metabolic analyses in neuroblastoma cells to investigate the effects of NCT-503 treatment on cellular metabolism. Glucose-derived carbon usage was traced using pulsed stable isotope labelling with ^13^C-glucose followed by quantitative metabolomics analysis. Incorporation of glucose-derived carbons into serine was significantly reduced upon NCT-503 treatment of BE(2)-C cells (high-level PHGDH), but not detectable in SH-EP cells (low-level PHGDH, Figure 2(A)), demonstrating that on-target effects were sensitive to the target level in the cells. NCT-503 treatment did not modulate incorporation of glucose-derived carbons into pyruvate in either cell line (Figure 2(A)). However, significantly less ^13^C-glucose was incorporated into citrate upon NCT-503 treatment of BE(2)-C and SH-EP cells compared to cells treated with the inactive drug control (Figure 2(B)). Incorporation of glucose-derived carbons into malate was significantly enhanced (Figure 2(B)), while acetyl-CoA levels remained stable after NCT-503 treatment in BE(2)-C and SH-EP cells compared to the respective controls (Figure 2(C)). These data demonstrate that besides the on-target inhibition of PHGDH directing glucose-derived carbons into serine, NCT-503 also reduces conversion of pyruvate into citrate while elevating the conversion of pyruvate into malate.

### Reduced carbon flow into the TCA cycle is an off-target NCT-503 effect

**Figure 3.**
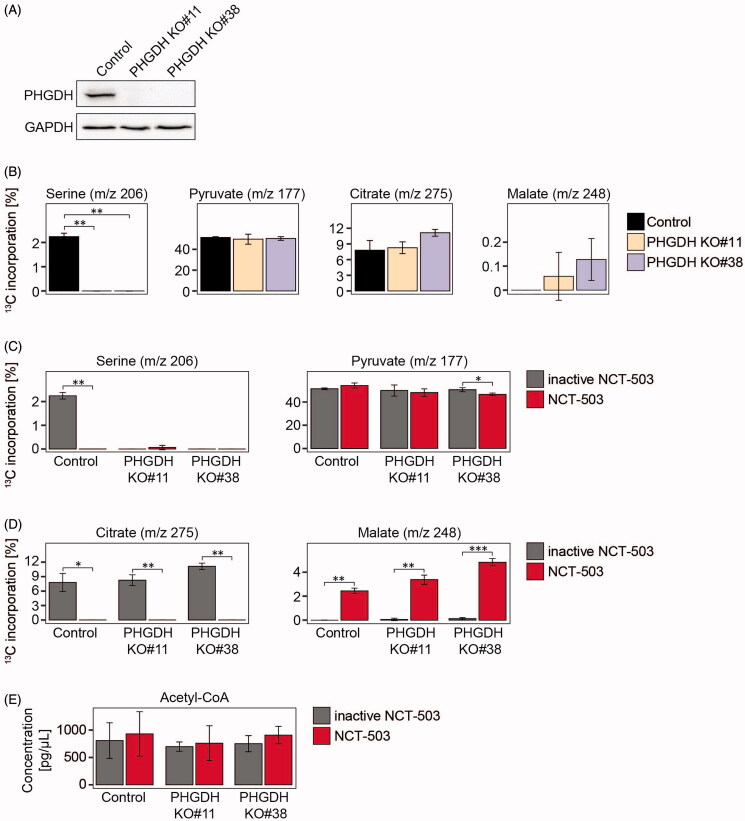
Altered carbon flow into the TCA cycle is an off-target effect of NCT-503. (A) Western blot analysis of PHGDH expression in PHGDH knockout clones #11 and #38. GAPDH served as loading control. (B) PHGDH knockout clones #11 and #38 and the respective control cells were seeded and maintained in medium containing fully labelled ^13^C glucose (13 mM) for 10 min before harvest. Shown are the percentages of ^13^C-label incorporation into serine, pyruvate, citrate and malate. (C–E) PHGDH knockout clones #11 and #38 and the respective control cells were seeded, cultured for 48 h with 10 µM NCT-503 or the inactive drug control and maintained in medium containing fully labelled ^13^C glucose (13 mM) for 10 min before harvest. Shown are the percentages of ^13^C-label incorporation into serine and pyruvate (C), citrate and malate (D) and the quantification of acetyl-CoA levels (E). *p < 0.05; **p ≤ 0.01; ***p ≤ 0.001.

To test whether the lack of efficient conversion of pyruvate into citrate was due to the NCT-503 compound rather than PHGDH inhibition, we turned to our two PHGDH knockout cell clones generated using CRISPR/Cas9 technology from the BE(2)-C cell line. Knockout of PHGDH enzyme activity was validated on the protein level (Figure 3(A)) and in the cell clone metabolome (Figure 3(B))^5^. PHGDH knockout did not affect incorporation of glucose-derived carbons into pyruvate, citrate or malate (Figure 3(B)). Glucose-derived carbons were similarly incorporated into pyruvate in PHGDH knockout clones treated with either NCT-503 or the inactive drug control (Figure 3(C)). However, NCT-503 treatment diminished glucose-derived ^13^C-incorporation into citrate, in line with considerably higher label incorporation into malate and the stable acetyl-CoA levels (Figure 3(D,E)). We conclude that NCT-503 triggers metabolic remodelling in neuroblastoma cells, independent of PHGDH expression.

### Off-target activity is not acting via citrate synthase

**Figure 4.**
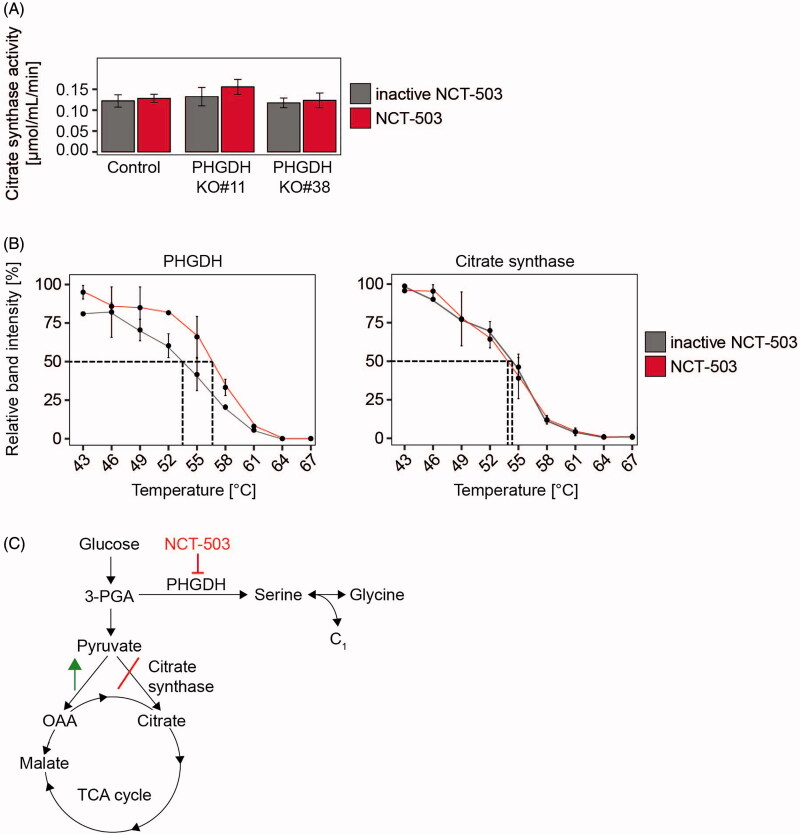
Citrate synthase activity and protein stability are not altered by NCT-503 treatment in neuroblastoma cells. (A) PHGDH knockout clones #11, #38 and the respective control cells were seeded, treated with 10 µM NCT-503 or inactive drug control for 24 h and assessed for citrate synthase activity. (B) Wildtype BE(2)-C cells were seeded, treated with 10 µM NCT-503 or inactive drug control for 48 h and subjected to cellular thermal shift assays followed by western blotting of PHGDH and citrate synthase expression. (C) Schematic model summarising the NCT-503 treatment-mediated effects in neuroblastoma cells.

We next investigated whether rerouteing glucose-derived carbons into the TCA cycle is based on NCT-503-mediated inhibition of citrate synthase, which converts oxaloacetate and acetyl-CoA into citrate. Citrate synthase activity was colorimetrically assessed in whole-cell lysates of the both PHGDH knockout clones and the BE(2)-C cell line. No significant changes in citrate synthase activity were measured between extracts from knockout clones and the parental cell line or between extracts from cultures treated with NCT-503 or the inactive drug control (Figure 4(A)). We also assessed target engagement by the drug using the cellular thermal shift assay^11^, which assesses thermal stabilisation of proteins caused or changed by drug binding. The melting curves represent the relative protein band intensities of PHGDH and citrate synthase after treatment with NCT-503 or the inactive drug control in BE(2)-C cell lysates as a function of temperature, and demonstrated a thermal shift in PHGDH but not in citrate synthase (Figure 4(B)). The clear shift was between the NCT-503-PHGDH aggregation temperature of 56.5 °C and the inactive drug control-PHGDH aggregation temperature of 53.5 °C (Figure 4(B)). The NCT-503-citrate synthase aggregation temperature was 54 °C while the inactive drug control-PHGDH aggregation temperature was 54.5 °C, thus, indicating no major changes in thermal stability of citrate synthase by NCT-503 treatment (Figure 4(B)). Our data demonstrate a novel mode of action for NCT-503, which limits glucose-derived carbon entry into citrate from pyruvate in neuroblastoma cells (Figure 4(C)). We propose that cells may adapt to this metabolic alteration by increased routeing of glucose-derived carbons from pyruvate into malate as an alternative pathway to support glucose-derived TCA cycle anaplerosis.

## Discussion

The current state of knowledge about the small molecule inhibitor, NCT-503, is that it selectively inhibits PHGDH, the rate-limiting enzyme in glucose-derived serine synthesis and is used to study serine synthesis through PHGDH in various cancer entities^3^^,^^13^. We identified an off-target effect of NCT-503 in a pulsed stable isotope-resolved metabolomics approach^6^ utilising ^13^C-glucose in neuroblastoma cell lines and genetically engineered *PHGDH* knockout clones. NCT-503 strongly reduced glucose-derived citrate synthesis in neuroblastoma cell lines, in line with increased incorporation of glucose-derived carbons entering the TCA cycle from pyruvate into malate. Carbon flow into the TCA cycle was independent of PHGDH level in the cells. This observed off-target effect was not acting via alterations in citrate synthase activity, but the exact mechanism employed in this off-target effect remains enigmatic.

NCT-503 treatment reduced the number of viable SH-EP and SK-N-AS cells expressing low-level PHGDH by up to 50% compared to cells treated with inactive drug control in our study. Similarly, Xia et al. recently reported that treating SK-N-AS and SH-SY5Y neuroblastoma cell lines, both with low-level PHGDH expression, with 10 µM NCT-503 reduced proliferation by up to 20% compared to controls^3^. Pacold et al. also described an anti-proliferative effect for the same NCT-503 concentration when treating a metastatic breast cancer cell line with low-level PHGDH expression, MDA-MB-231^4^. This response pattern in additional cell lines with low target levels obtained in two independent studies, which is characterised by a comparatively weaker but detectable anti-proliferative effect, corroborates our own data and may be explained by our observed off-target effect of NCT-503. However, NCT-503 treatment did not reduce proliferation in the ZR-75-1 breast carcinoma cell line and the SK-MEL-2 malignant melanoma cell line, which both express low PHGDH levels^4^, suggesting that phenotypic consequences of the off-target effect described here vary among cell lines. Cellular metabolism is, among other factors, highly influenced by its genetic background^14^. Individual oncogenes and tumour suppressors specifically influence metabolic circuitry^15^, and could compensate for the NCT-503 treatment-mediated reduction in glucose-derived citrate synthesis so that fundamental processes in cancer cells, such as proliferation, are not detectably altered. Cancer cells are characterised by a high level of metabolic adaptability, and interact with non-transformed cells within their respective microenvironment to survive and proliferate in poorly perfused niches where nutrients are rare^16^. Data showing that the low-level PHGDH expressing MDA-MB-231 cells, which were weakly responsive to NCT-503 treatment *in vitro,* were non-responsive when grown as xenografted tumours in mice^4^ points towards the importance of the tumour microenvironment and should be investigated in future studies. Altogether, the off-target effect described in this study might explain the sensitivity of certain cells expressing low PHGDH levels towards NCT-503 treatment.

By applying pulsed stable isotope-resolved metabolomics, we observed a decrease in glucose-derived carbons into serine as well as a rerouteing of glucose-derived carbons entering the TCA cycle in neuroblastoma cells treated with NCT-503. While blockage of glucose-derived carbon incorporation into serine and glycine in NCT-503-treated cells has already been studied in detail^3^^,^^4^, conversion of glucose-derived carbons into TCA cycle intermediates under NCT-503 treatment has not been addressed in detail. Reid et al. used kinetic flux profiling^17^^,^^18^ with ^13^C-glucose labelling to directly measure flux into metabolic pathways, and demonstrated that 25 µM NCT-503 reduced flux through the pentose phosphate pathway and TCA cycle, including a reduced fraction into citrate/isocitrate, in epithelial cancer cells^13^. Treatment of these cells with another PHGDH inhibitor, PKUMDL-WQ-2101^19^, induced similar reductions in metabolic flux. *PHGDH* knockout clones were used to confirm that the observed kinetic profiles upon PKUMDL-WQ-2101 treatment were not due to off-target effects^13^. However, kinetic flux profiling in NCT-503 treated *PHGDH* knockout clones has not been reported^13^. Our data obtained with NCT-503-treated *PHGDH* knockout neuroblastoma cell clones demonstrates that the reduced incorporation of glucose-derived carbons into citrate is completely independent of the expression of PHGDH itself and, thus, due to an off-target effect of NCT-503. Future studies with newly emerging PHGDH inhibitors are required to investigate whether the reduced flux into citrate/isocitrate observed with PKUMDL-WQ-2101^13^ represents a common on-target effect of the class of PHGDH inhibitors.

We have shown that ^13^C-glucose incorporation rates into pyruvate were similar between cells treated with NCT-503 or the inactive drug, but incorporation rates into citrate were significantly reduced in all NCT-503-treated cell lines. Citrate synthase catalyses the first step of the TCA cycle^20^ and is upregulated in several cancer types^21^^,^^22^. Since pyruvate is converted into acetyl-CoA and subsequently into citrate via the pyruvate dehydrogenase complex and citrate synthase, we analysed whether citrate synthase could be a possible NCT-503 target. NCT-503, surprisingly, did not alter citrate synthase activity *in vitro* or the thermal stability of the enzyme, indicating that the reduction in glucose-derived carbon incorporation into citrate induced by NCT-503 treatment is not caused by direct inhibition of citrate synthase activity or stable binding by NCT-503 that alters the thermodynamic properties of the enzyme. Thus, the underlying mechanism remains unclear. Besides diminished routeing of glucose-derived carbons from pyruvate into citrate, we observed enhanced routeing of glucose-derived carbons from pyruvate into malate in BE(2)-C and SH-EP cells as well as two *PHGDH* knockout clones by NCT-503 treatment. These data suggest a shift from citrate synthase to enhanced usage of the pyruvate carboxylase pathway for TCA cycle anaplerosis. Pyruvate carboxylase requires acetyl-CoA as an allosteric activator^23^ that was expressed at steady levels in our NCT-503-treated cells. We propose that NCT-503 treatment enhances pyruvate carboxylase pathway activity in neuroblastoma cells as an alternative route to fuel the TCA cycle with glucose-derived carbons. Activation of pyruvate carboxylase was first described by Fan et al. in human non-small cell lung cancer^24^. Cheng et al. found that glutaminase-silenced glioblastoma cells induce a compensatory anaplerotic mechanism catalysed by pyruvate carboxylase to use glucose-derived pyruvate rather than glutamine for anaplerosis^25^, thus, demonstrating the high flexibility of cancer cells to circumvent perturbations in their metabolic networks.

Off-target effects are a common theme in pharmacology. For example, the MEK-inhibitors, PD98059 and U0126, were recently reported not only to inhibit calcium entry into cells but also affect AMPK activity independent of their on-target effect on MEK1/2^26^^,^^27^. Nevertheless, these compounds are intensely studied in clinical trials and continue to be routinely used in preclinical models to pharmacologically modulate MAPK/ERK signalling. The small molecule inhibitor, NCT-503, not only targets PHGDH, the rate limiting enzyme in *de novo* serine synthesis, but also induces a remodelling of glucose-derived carbon flow into the TCA cycle independently of PHGDH expression in neuroblastoma cells. At this stage, we can only speculate whether the substantial anti-tumour activity reported by us and others in preclinical models of several cancer types could at least partly be due to reduced incorporation of glucose-derived carbons into citrate. The data presented here emphasises the importance to preclinically characterise the molecular actions of NCT-503 in detail in the potential target cancers of interest in future studies.

## Supplementary Material

Supplemental Material

## Data Availability

The authors confirm that the data supporting the metabolomics findings of this study are available within the article supplementary materials. Other data will be made available upon request.
